# Benzylamines as highly potent inhibitors of the sterol biosynthesis pathway in *Leishmania amazonensis* leading to oxidative stress and ultrastructural alterations

**DOI:** 10.1038/s41598-022-15449-3

**Published:** 2022-07-04

**Authors:** Sara Teixeira de Macedo-Silva, Gonzalo Visbal, Gabrielle Frizzo Souza, Mayara Roncaglia dos Santos, Simon B. Cämmerer, Wanderley de Souza, Juliany Cola Fernandes Rodrigues

**Affiliations:** 1 Laboratório de Ultraestrutura Celular Hertha Meyer, Instituto de Biofísica Carlos Chagas Filho, Universidade Federal do Rio de Janeiro, Rio de Janeiro, Brazil; 2 Instituto Nacional de Ciência e Tecnologia de Biologia Estrutural e Bioimagem, Rio de Janeiro, Brazil; 3 Centro Nacional de Biologia Estrutural e Bioimagem, CENABIO, UFRJ, Rio de Janeiro, Brazil; 4 Instituto Nacional de Metrologia, Qualidade e Tecnologia, Inmetro, Brazil; 5 Instituto de Química, Departamento de Química Orgânica, UNICAMP, Campinas, Brazil; 6 Núcleo Multidisciplinar de Pesquisa em Biologia, Divisão Biologia (NUMPEX-BIO), Campus UFRJ-Duque de Caxias Prof. Geraldo Cidade, Universidade Federal do Rio de Janeiro, Rodovia Washington Luiz, n. 19.593, km 104.5-Santa Cruz da Serra, Duque de Caxias, RJ 25.240-005 Brazil

**Keywords:** Drug discovery, Parasitology

## Abstract

Leishmaniasis is a neglected disease caused by protozoan parasites of the *Leishmania* genus. Benzylamines are a class of compounds selectively designed to inhibit the squalene synthase (SQS) that catalyzes the first committed reaction on the sterol biosynthesis pathway. Herein, we studied seven new benzylamines (SBC 37–43) against *Leishmania amazonensis*. After the first screening of cell viability, two inhibitors (SBC 39 and SBC 40) were selected. Against intracellular amastigotes, SBC 39 and SBC 40 presented selectivity indexes of 117.7 and 180, respectively, indicating high selectivity. Analysis of the sterol composition revealed a depletion of endogenous 24-alkylated sterols such as episterol and 5-dehydroepisterol, with a concomitant accumulation of fecosterol, implying a disturbance in cellular lipid content. This result suggests a blockade of de novo sterol synthesis at the level of SQS and C-5 desaturase. Furthermore, physiological analysis and electron microscopy revealed three main alterations: (1) in the mitochondrion; (2) the presence of lipid bodies and autophagosomes; and (3) the appearance of projections in the plasma membrane. In conclusion, our results support the notion that benzylamines have a potent effect against *Leishmania amazonensis* and should be an exciting novel pharmaceutical lead for developing new chemotherapeutic alternatives to treat leishmaniasis.

## Introduction

Leishmaniasis is an endemic neglected disease caused by several species of *the Leishmania* genus. The leishmaniasis transmission was reported in 98 countries and 3 territories on 5 continents. The estimated world prevalence for all clinical manifestations of the disease is 12 million, with 58,000 visceral leishmaniasis (VL) cases and 220,000 cutaneous leishmaniasis (CL) cases per year^1,2^. Multiple species, including *L. amazonensis* and *L. braziliensis*, are the causative agents of cutaneous leishmaniasis (CL) in the New World. Furthermore, *L. amazonensis* can also cause mucocutaneous leishmaniasis (MCL), which results in progressive destruction of the naso-oropharyngeal mucosa, and diffuse cutaneous leishmaniasis (DCL), the most severe cutaneous form of the disease that is characterized by a diffuse infiltration in the skin of the patients and highly resistant to all kind of chemotherapy available^3,4^. Besides the severe clinical manifestations of leishmaniasis, few drugs are available for its treatment.

Pentavalent antimonials have been the first treatment choice for more than 70 years in some countries worldwide, despite the parenteral route of administration, high toxicity, and cost^5^. Second-line treatments are based on the use of amphotericin B formulations and pentamidine. In some countries in Asia, Africa, and Europe, miltefosine (Impavido) is currently the first-line treatment^5,6^. Among the chemotherapeutic agents available, miltefosine is the only orally available treatment; however, it is teratogenic and not indicated for women of fertile age^7,8^. None of the available drugs can be considered ideal due to their high toxicity, long duration of treatment, and severe side effects, which often lead to treatment abandonment. Furthermore, these treatments do not eliminate the parasite in the infected individuals^9,10^.

Trypanosomatids and fungi have an endogenous requirement of ergosterol and other 24-alkylated sterols for growth and survival, which are absent in mammal cells. Thus, the enzymes involved in the sterol biosynthesis pathway are interesting targets for new treatments, and several works have shown the effect of different sterol biosynthesis inhibitors (SBIs) in trypanosomatids^11–17^. Benzyl farnesyl amine mimetics are one class of selective inhibitors of the squalene synthase (SQS), an important enzyme that catalyzes the condensation of two molecules of farnesyl pyrophosphate to produce squalene. SQS catalyzes the first committed step in sterol biosynthesis, and its inhibition does not affect the synthesis of isoprenoids, which are also essential molecules for eukaryotic cells. For years, the research for specific squalene synthase inhibitors (SQSi) has involved intensive efforts of industrial and academic investigators because of the potential use of these inhibitors in the treatment of coronary heart disease and hypercholesterolemia^18–20^.

Among the selective inhibitors of the SQS^21–23^, we early investigated the antiprotozoal potential of BPQ-OH (3-biphenyl-4-yl-3-hydroxyquinuclidine), an arylquinuclidine, with significant biological activity against *T. cruzi* and *L. mexicana*, resulting in growth inhibition, cell lysis, and complete depletion of endogenous squalene and 24-methyl sterols^27^. Against *L. amazonensis*, BPQ-OH presented a potent growth inhibition effect against both developmental stages of the parasite leading to several changes in the ultrastructure of promastigotes^28^. Furthermore, the other two quinuclidine derivatives, E-5700 and ER-119884, also showed potent antiproliferative effects against *T. cruzi*^30^ and *L. amazonensis*, also in combination with C14α-demethylase inhibitors [azoles]^17,23^.

This work evaluated the effect of another class of selective SQS inhibitors, the novel mimetics of benzyl farnesyl amine, against *Leishmania amazonensis*. Several aspects of the anti-*Leishmania* activity of these compounds were investigated at different times of treatment; we studied the antiproliferative, ultrastructural, and biochemical effects induced by them. Moreover, we found one derivative that is among the most selective compounds for the parasite with low toxicity for the mammalian cells.

## Material and methods

### Parasites

The MHOM/BR/75/Josefa strain of *Leishmania amazonensis* used in this study was gently provided by the *Leishmania* Collection of the Instituto Oswaldo Cruz (code IOCL 0071-FIOCRUZ). It has been maintained via inoculation into the base of BALB/c mouse tails. Amastigotes were obtained from the lesions of infected mice and transformed into promastigotes that were maintained in Warren’s medium [brain heart infusion plus hemin and folic acid]^24^ supplemented with 10% fetal bovine serum (FBS) at 25 °C. Infective metacyclic promastigotes were used to obtain intracellular amastigotes in macrophage culture. Firstly, peritoneal macrophages from BALB/c mice were harvested by washing them with Hanks’ solution and plated in 24-well tissue culture chamber slides, allowing them to adhere to the slides for 24 h in RPMI medium (Gibco) supplemented with 10% FBS at 37 °C in 5% CO_2_. After this, adherent macrophages were infected with metacyclic promastigotes at a macrophage-to-parasite ratio of 1:10 at 35 °C in 5% CO_2_ for 2 h. These cultures were maintained for 24 h in RPMI medium supplemented with 10% FBS for the assays with intracellular amastigotes.

### Drugs

**Figure 1:**
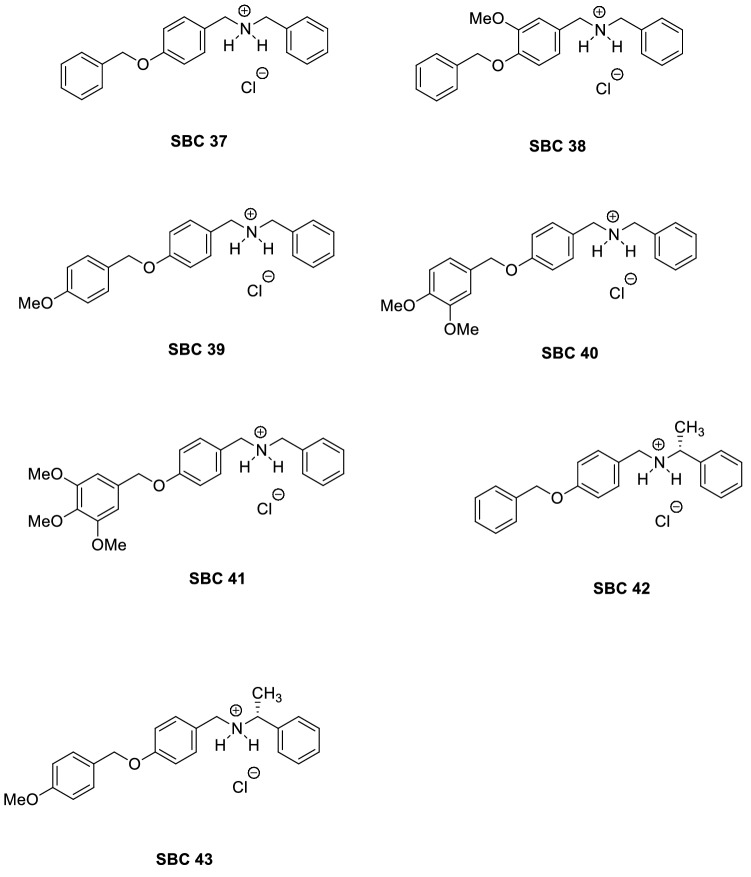
Benzyl farnesyl amine mimetics, SBC 37–43, used in this study.

The benzyl farnesyl amine mimetics, SBCs 37–43, were prepared by chemical synthesis at IQ-UNICAMP according to an experimental procedure previously described by Cämmerer and Souza^25^. Structures of those *N*-[4-[benzyloxy] benzyl]-benzene-methaneamine derivatives, SBCs 37–43, are displayed in Fig. 1. These compounds have been recently reported to exhibit significant biological activity against intracellular amastigotes of *Trypanosoma cruzi*^25^. Compounds were used as hydrochloride salts, purified by one recrystallization from analytical grade ethanol, and dried in a high vacuum at room temperature.

### Cell viability and cytotoxicity assays

For primary screening of the antileishmanial effects of the SBCs 37–43, we evaluated the cell viability and cytotoxicity effects in *L. amazonensis* promastigotes and peritoneal macrophages using the CellTiter 96^®^ Aqueous MTS Assay (Promega, United States)^16,29^. For analysis in promastigotes, we started the culture at a cell density of 1 × 10^6^ cells/ml in Warren’s medium supplemented with 10% FBS. After 24 h, different concentrations of SBC 37–43 were added to the cultures. Cell viability and the cytotoxic effects were measured at 24, 48, and 72 h of treatment, when all groups, including untreated, were transferred to a transparent 96-well plate in triplicate. MTS/PMS assay reaction was quantified by optical density measurement at 490 nm in a microplate reader and SpectraMax M_2_/M_2_^e^spectrofluorometer (Molecular Devices, United States). As a negative control, parasites were fixed with 0.4% nascent formaldehyde for 10 min at room temperature before the incubation. Cytotoxicity effects of SBCs 37–43 in murine macrophages were also evaluated using the same MTS/PMS assay reaction described above. After washing the peritoneal cavity of the BALB/c mice with Hanks's solution, murine macrophages were obtained and cultivated in a transparent 96-well plate with RPMI medium supplemented with 10% FBS and maintained at 37 °C in 5% CO_2_. After 24 h of cultivation, SBCs 37–43 were added at different concentrations. Macrophage viability was measured at 24, 48, and 72 h of treatment. MTS/PMS assay reaction was also quantified by optical density measurement at 490 nm in a microplate reader and SpectraMax M_2_/M_2_^e^spectrofluorometer (Molecular Devices, United States). The cytotoxicity concentration to reduce 50% of viable macrophages (CC_50_) was determined.

### Growth inhibition of promastigotes and amastigotes of *L. amazonensis*

After evaluating the cell viability and cytotoxic effects by MTS assay in promastigote forms, we also analyzed the effects of the SBCs 37–43 in the growth of promastigotes. For this, promastigote cultures were initiated at a cell density of 1.0 × 10^6^ cells/ml. After 24 h of growth, SBCs 37–43 were added at different concentrations from concentrated stock solutions, and cell densities were evaluated daily over 96 h of growth using a Neubauer chamber. Based on the analysis of CC_50_ and IC_50_ in promastigotes, and the cytotoxic effects in murine macrophages, three benzyl farnesyl amine mimetics (SBCs 37, 39, and 40) were chosen for evaluation against intracellular amastigotes. To evaluate the effects of compounds on *L. amazonensis* intracellular amastigotes, macrophages were infected as described previously and incubated with different concentrations of compounds after 24 h of infection. Fresh medium was added daily until 3 days (24, 48, and 72 h of treatment). After this time, cultures were fixed in Bouin’s solution^17^, washed with 70% ethanol, distilled water, and then stained with Giemsa solution for 1 h. The number of intracellular amastigotes was obtained after the count in light microscopy. Association indexes ((mean number of parasites internalized X percentage of infected macrophages)/total number of macrophages) were determined and used as a parameter to calculate the percentage of infection for each condition used in this study. The concentration that inhibited 50% of growth (IC_50_) and selective index (SI) was calculated. The CC_50_ and IC_50_ were calculated by fitting the values to a non-linear curve analysis. The regression analyses were performed with SigmaPlot 10 software.

### Estimation of the mitochondrial transmembrane electric potential

Mitochondrial transmembrane electric potential (Δψ_*m*_) of the untreated and treated promastigotes was analyzed using the JC-1 fluorochrome (Molecular Probes, United States). This lipophilic and cationic mitochondrial vital dye accumulates in the mitochondria in response to Δψ_*m*_ since its fluorescence indicates an energized mitochondrial state^15^. JC-1 exists as a J-monomer that, in the absence of Δψ_*m*_, accumulates in low concentration with emission wavelength at 530 nm (green fluorescence); however, in the presence of Δψ_*m*_, JC-1 accumulates as J-aggregates with emission at 590 nm (red fluorescence). Parasites were prepared as previously described^15,16^. For each sample, 1 × 10^7^ parasites were incubated with 10 μg/ml JC-1 for 25 min, with readings made every minute using a microplate reader and spectrofluorometer SpectraMax M2/M2^e^ (Molecular Devices, United States). As a positive control for the mitochondrial membrane depolarization, cells were incubated with 2 μM FCCP (a mitochondrial protonophore) during the initial 20 min of reading. After 20 min, 2 μM FCCP was added to all samples to abolish the Δψ_*m*_. The relative Δψ_*m*_ values were obtained, calculating the ratio between the reading at 590 nm and 530 nm (590:530 nm). Experiments were independently repeated at least three times in triplicate, and the graphic shows the mean and standard deviation of one representative experiment.

### Evaluation of ROS production

As described previously, intracellular ROS levels were evaluated in control and compound-treated promastigotes^17^. For this, 3 × 10^7^ promastigotes were harvested, washed twice in PBS (pH 7.2), and incubated with 10 µg/ml H_2_DCFDA [a cell-permeable green probe; Molecular Probes, United States] in PBS for 1 h at 25 °C. After 1 h, cells were washed and resuspended in PBS, added to a black 96-well plate, and then analyzed in a microplate reader and spectrofluorometer SpectraMax M2/M2^e^ (Molecular Devices, United States), using the pair of 507 nm and 530 nm wavelengths as emission and excitation wavelengths, respectively.

### Lipid bodies accumulation

For analysis of lipid bodies accumulation, 1.0 × 10^7^ cells were harvested, washed in PBS (pH 7.2), and incubated with 10 µg/ml Nile Red (Sigma, Brazil) for 20 min. After this step, cells were washed twice in PBS, resuspended in 200 µl of PBS, and then added to a black 96-well plate. Readings were taken in a microplate reader and spectrofluorometer SpectraMax M2/M2^e^ (Molecular Devices, United States), using the wavelengths 485 and 538 nm for excitation and emission, respectively.

### Electron microscopy

First, control and treated promastigotes and intracellular amastigotes were fixed in 2.5% glutaraldehyde in 0.1 M cacodylate buffer (pH 7.2) for 1 h at room temperature. Second, the samples were postfixed in a solution containing 1% OsO_4_, 1.25% potassium ferrocyanide, and 5 mM CaCl_2_ in 0.1 M cacodylate buffer (pH 7.2) for 30 min. For scanning electron microscopy, promastigotes were dehydrated in ethanol (30, 50, 70, 90, and 100%) and critical point-dried in CO_2_. After that, samples were sputtered with a thin gold layer and observed under a FEI Quanta 250 scanning electron microscope. For transmission electron microscopy, cells were dehydrated in acetone and embedded in epoxy resin. After that, ultrathin sections were stained with uranyl acetate and lead citrate and observed under a Zeiss 900 electron microscope.

### Electron tomography

For electron tomography, ribbons of 200 nm thick serial sections were obtained by ultrathin sectioning in an ultramicrotome. These ribbons were collected in formvar-coated copper slot grids. After that, colloidal gold particles (10 nm) were deposited on both surfaces of the sections, being used as fiducial markers for the alignment of the tilted views. Single-axis tilt series (± 60° with 1° increment) were produced from samples using Xplore3D software and a Tecnai-G2 (FEI Company, Eindhoven, Netherlands) electron microscope operating at 200 kV. 3D reconstruction was performed using the IMOD software package^27^. Furthermore, tomogram generation by R-weighted back-projection was performed using ETOMO, and virtual slices were manually segmented using 3DMOD, which was also used to produce 3D models.

### Extraction, separation of neutral lipids, and free sterol analysis

Total lipids were extracted from control and drug-treated *Leishmania amazonensis* promastigotes to analyze the effects of SBC 39 and SBC 40 on the free sterol composition of the promastigotes, as described previously^17,28^. Neutral lipids were analyzed by MS, and mass spectra were obtained by electron ionization (EI) at 70 eV according to the protocol published previously^17,28^. The assignment of structures was based on relative chromatographic behaviors, as well as the characteristic fragmentation patterns in MS, and by comparison of the mass spectra with those available in the National Institute of Standards and Technology (NIST) Research Library located at the NIST Mass Spectrometry Data Center.

### Calibration for cholesterol and ergosterol determination

Five calibration standards were prepared from the pure standard of cholesterol, ergosterol, β-sitosterol, stigmasterol, and 5α-ergosta-8(14)-en-3β-ol purchased from Sigma-Aldrich Co. Different calibration solutions were prepared using ethyl acetate as solvent. To quantify cholesterol and ergosterol, standards were used at different concentrations of 0.08, 0.10, 0.25, 0.50, and 1.0 mM to plot the standard curve. From each calibration solution, 1 μl was injected (run in triplicate) into the GC–MS system to achieve the regression plot of various concentrations versus their peak area. To estimate the level of endogenous sterol/cell in control and drug-treated cultures, the total areas of the corresponding chromatographic peaks were divided by the cell densities of the cultures.

### Statistical analysis

All the graphics in the figures were created using the means of three independent experiments, and the bars represent the standard deviations of the means. The statistical significance of differences among the groups was assessed using the one-way or two-way analysis of variance (ANOVA) test, followed by Bonferroni’s multiple-comparison test in the GraphPad Prism 5 software. Results were considered statistically significant when *P* was < 0.05(*), < 0.01(**), and < 0.001(***).

### Ethics approval

The experiments using BALB/c mice to isolate macrophages and to maintain *Leishmania* parasites were approved by the Ethics Committee for Animal Experimentation of the Health Sciences Centre, Federal University of Rio de Janeiro (Protocols n. IBCCF 096/097/106), according to the Brazilian federal law (11.794/2008, Decreto no 6.899/2009). Furthermore, all animals received humane care in compliance with the “Principles of Laboratory Animal Care” formulated by the National Society for Medical Research and the “Guide for the Care and Use of Laboratory Animals” prepared by the National Academy of Sciences, USA. The experiment involving animals follows the recommendations described in the ARRIVE guidelines.

## Results

### The cytotoxic and antiproliferative effects of benzyl farnesyl amine mimetics SBC 37–43 against macrophages and *Leishmania amazonensis*

**Figure 2:**
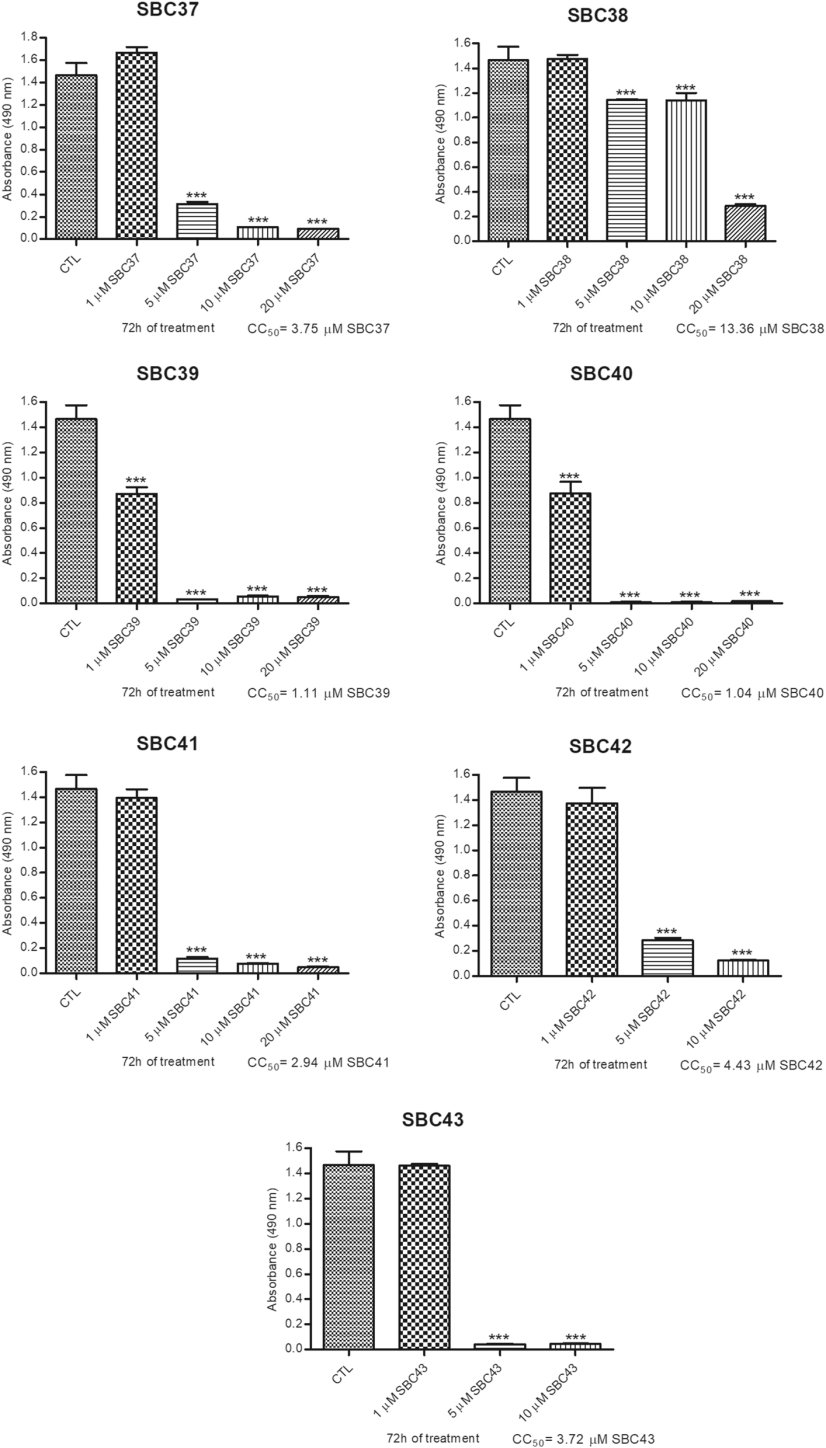
Evaluation of cytotoxicity effects of SBCs on *Leishmania amazonensis* promastigotes. Cell viability and cytotoxicity were evaluated against promastigotes using the MTS/PMS assay after 72 h of treatment. The cytotoxicity concentration to reduce 50% of viable promastigotes (CC_50_) was determined. SBC37, SBC39, SBC40, SBC41, SBC42, and SBC43 presented CC_50_ lower than 5 µM. Bars represent standard deviation; *p < 0.05, **p < 0.01, and ***p < 0.001.

**Figure 3:**
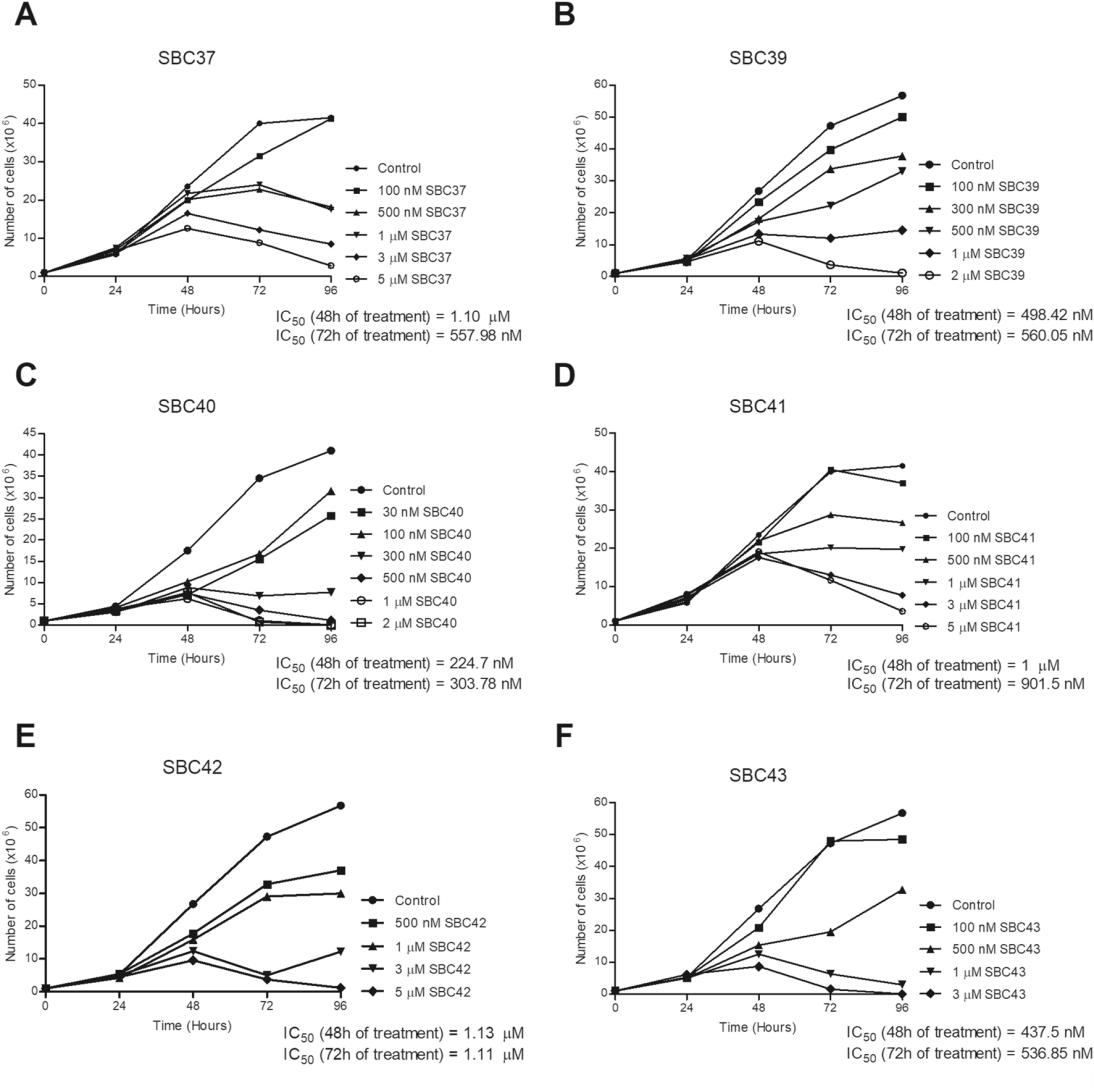
Evaluation of antiproliferative effects of SBCs on *Leishmania amazonensis* promastigotes. Parasites were treated with SBCs for 72 h to evaluate the parasite growth. The inhibitors were added at different concentrations after 24 h of growth. The most potent inhibitors were SBC 37 (**A**), SBC 39 (**B**), SBC 40 (**C**), and SBC 43 (**F**) with IC_50_ of 557 nM, 560.05 nM, 303.78 nM, and 536.85 nM, respectively, after 72 h of treatment.

Figure 2 shows the cytotoxic effects of seven benzyl farnesyl amine mimetics SBC 37–43 by MTS/PMS assay of *Leishmania amazonensis* promastigotes. Six of them had CC_50_ lower than 5 µM (SBC 37, SBC 39, SBC 40, SBC 41, SBC 42, and SBC 43), indicating an excellent effect against *Leishmania amazonensis*. After that, these six inhibitors were analyzed against *Leishmania amazonensis* promastigotes to evaluate their potential antiproliferative effects. Figure 3 shows the growth curves of the most potent inhibitors (SBC 37, 39, 40, 43), which presented IC_50_ values in the nanomolar range after 72 h of treatment [557 nM, 560.05 nM, 303.78 nM, and 536.85 nM, respectively].

**Figure 4:**
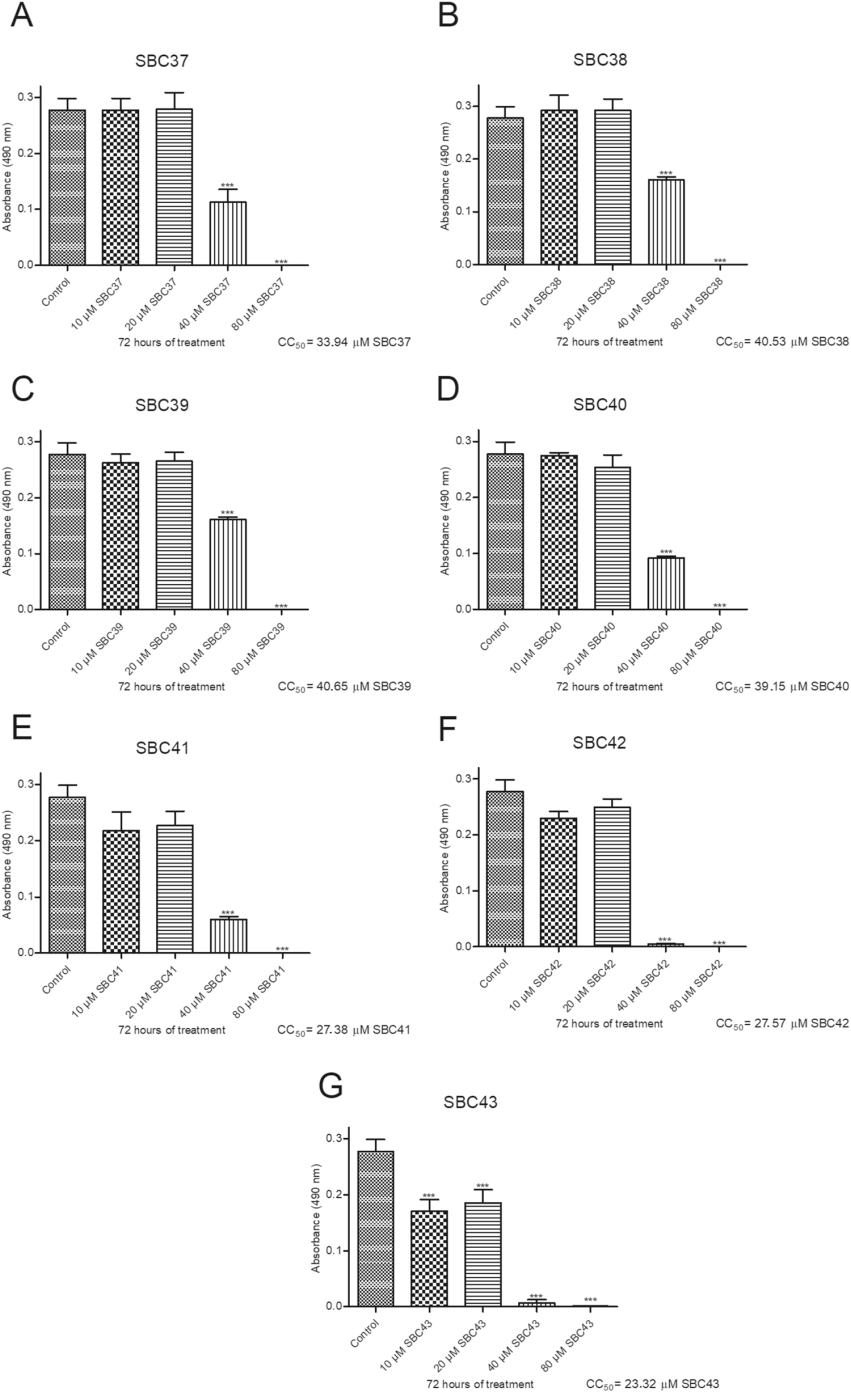
Evaluation of cytotoxicity effects of SBCs on murine macrophages. The MTS/PMS assay was used to evaluate the cytotoxicity against murine macrophages after 72 h of treatment. The compounds SBC 37 (**A**), SBC 38 (**B**), SBC 39 (**C**), and SBC 40 (**D**) presented low cytotoxicity to mammalian cells, with CC_50_ of 33.94 µM, 40.53 µM, 40.65 µM, and 39.15 µM, respectively. Bars represent standard deviation; *p < 0.05, **p < 0.01, and ***p < 0.001.

**Figure 5:**
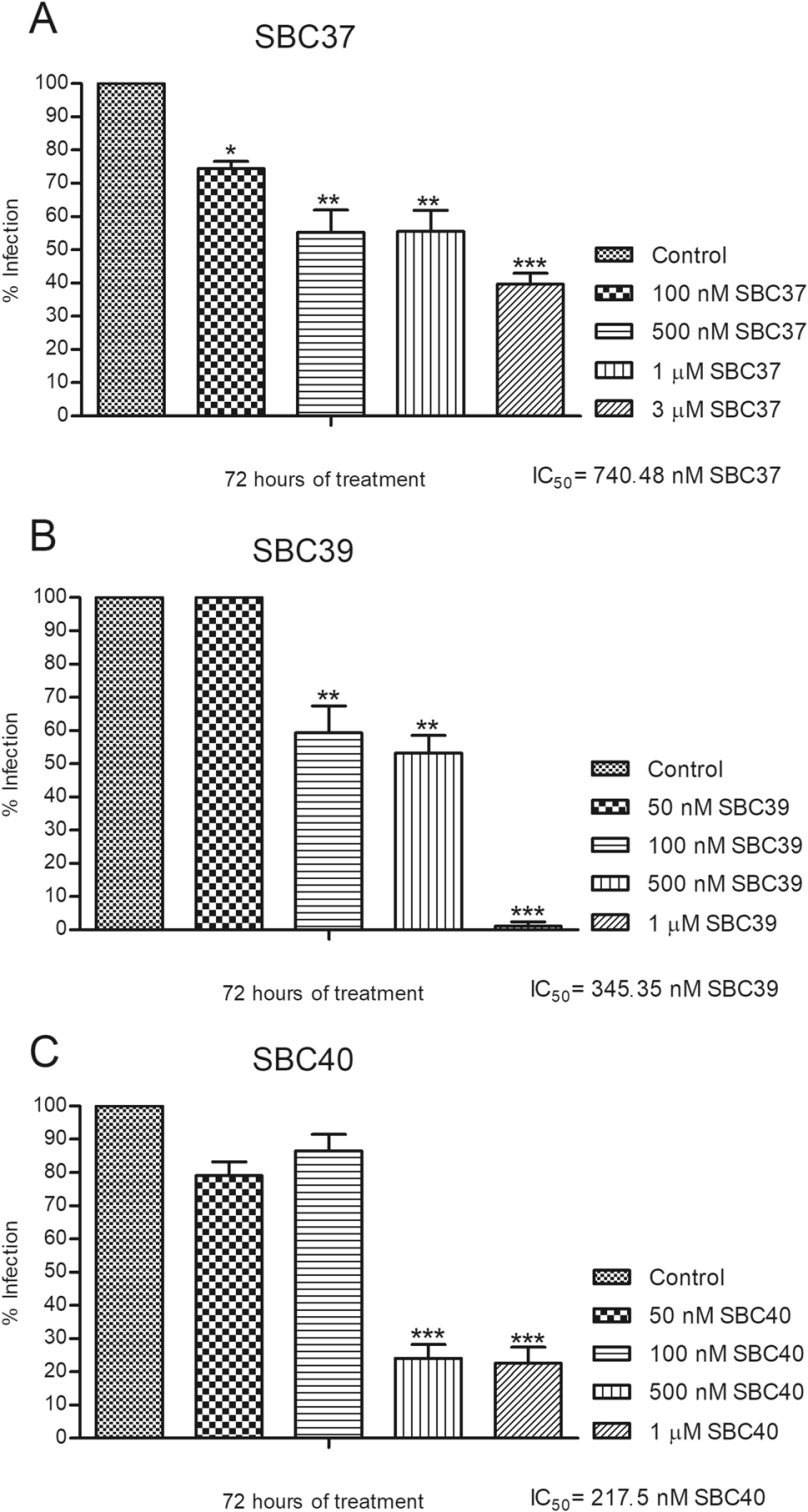
Evaluation of antiproliferative effects of SBC 37, SBC 39, and SBC 40 on *Leishmania amazonensis* intracellular amastigotes. Parasites were treated with different concentrations of the inhibitors for 72 h to evaluate the parasite growth. After 72 h of treatment, the IC_50_ was determined for each inhibitor tested, and the values were 740.48 nM, 345.35 nM, and 217.5 nM, respectively. Thus, the selectivity index obtained was 45.83, 117.7, and 180, respectively. Bars represent standard deviation; *p < 0.05, **p < 0.01, and ***p < 0.001.

We also evaluated the cytotoxic effects of the SBCs 37–43 against murine macrophages using MTS/PMS assay after 72 h of treatment. Figure 4 shows that SBC 37, 38, 39, and 40 presented low cytotoxicity to mammalian cells, with CC_50_ values of 33.94 µM, 40.53 µM, 40.65 µM, and 39.15 µM, respectively. Based on these results obtained for macrophages and promastigotes, we decided to evaluate the effects of three of them (SBCs 37, 39, and 40) against intracellular amastigotes. Figure 5 shows the antiproliferative effects of SBC 37, 39, and 40 against intracellular amastigotes after 72 h of treatment; all IC_50_ values found to them were in the nanomolar range (740.48 nM, 345.35 nM, and 217.5 nM, respectively) (Fig. 5A–C). Thus, the selectivity index obtained was 45.83, 117.7, and 180, respectively, after 72 h of treatment.

### SBC 39 and SBC 40 alter the morphology of promastigotes

**Figure 6:**
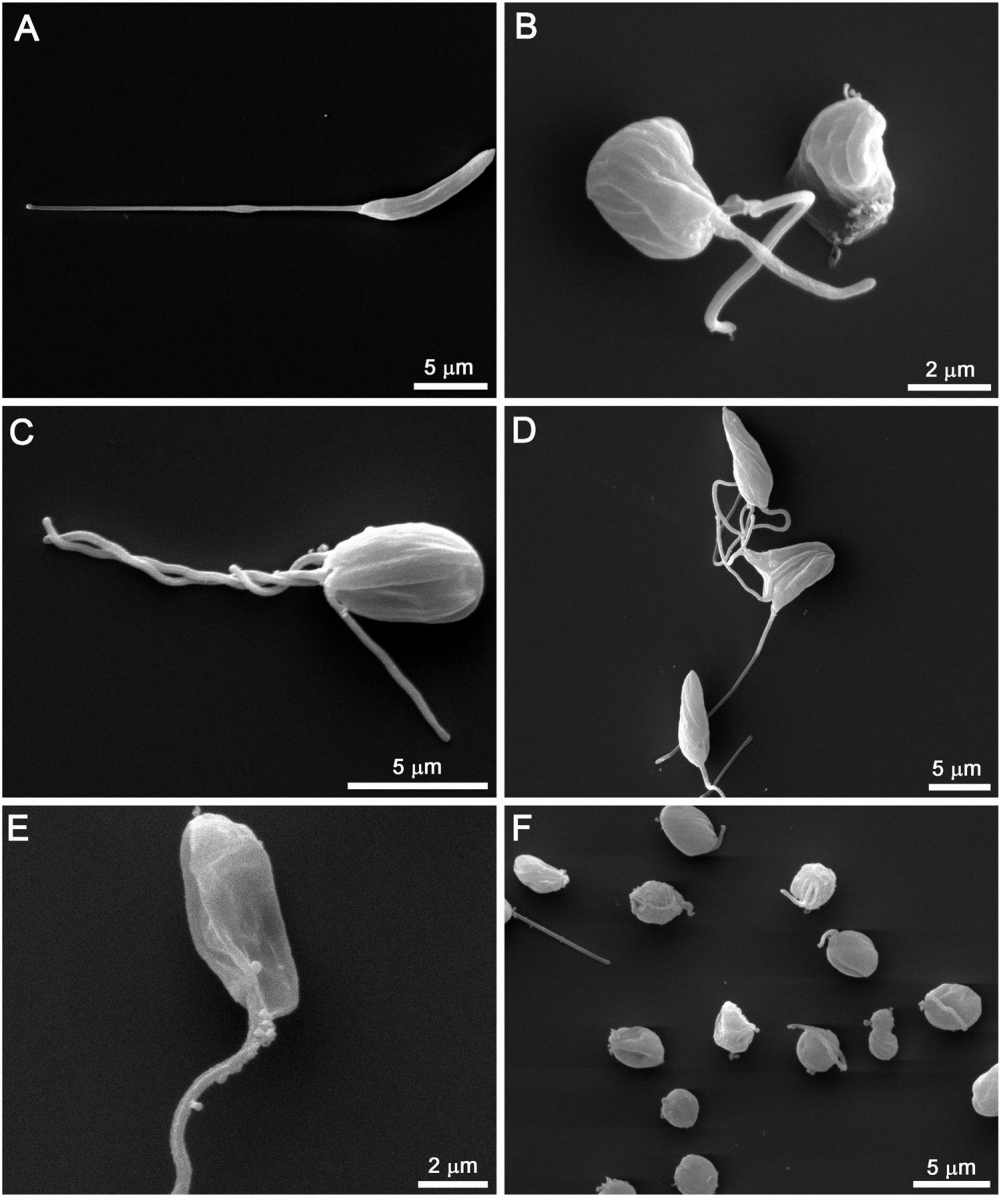
Scanning electron microscopy (SEM) of *L. amazonensis* promastigotes treated with SBCs for 48 h. (**A**) Control. (**B**,**C**) 500 nM SBC 39. (**D**) 1 µM SBC 39. (**E**) 100 nM SBC 40. (**F**) 300 nM SBC 40. The images show several changes in the morphology of the parasite. Promastigotes appeared rounded and swollen (**B**,**C**,**F**), with vesicles next to the flagellar pocket (**E**). Sometimes more than two flagella can be observed (**C**,**D**).

Scanning electron microscopy revealed important changes in the morphology of promastigotes treated with SBC 39 and SBC 40 for 48 h (Fig. 6A–F). Figure 6A shows a control *L. amazonensis* promastigote without any alteration in the morphology of the cell body, surface, and flagellum (Fig. 6A). Treatment with lower concentrations of SBCs induced several alterations, such as the presence of parasites rounded and swollen (Fig. 6B,C,F), also presenting vesicles budding from the region near the flagellar pocket (Fig. 6E) and sometimes more than two flagella (Fig. 6C,D). After 48 h of treatment with 300 nM SBC 40, all parasites were completely rounded (Fig. 6F). These results indicate the potent effect of these inhibitors on altering the morphology of promastigotes.

### SBC 39 and SBC 40 alter mitochondrion function and induce lipid bodies accumulation

**Figure 7:**
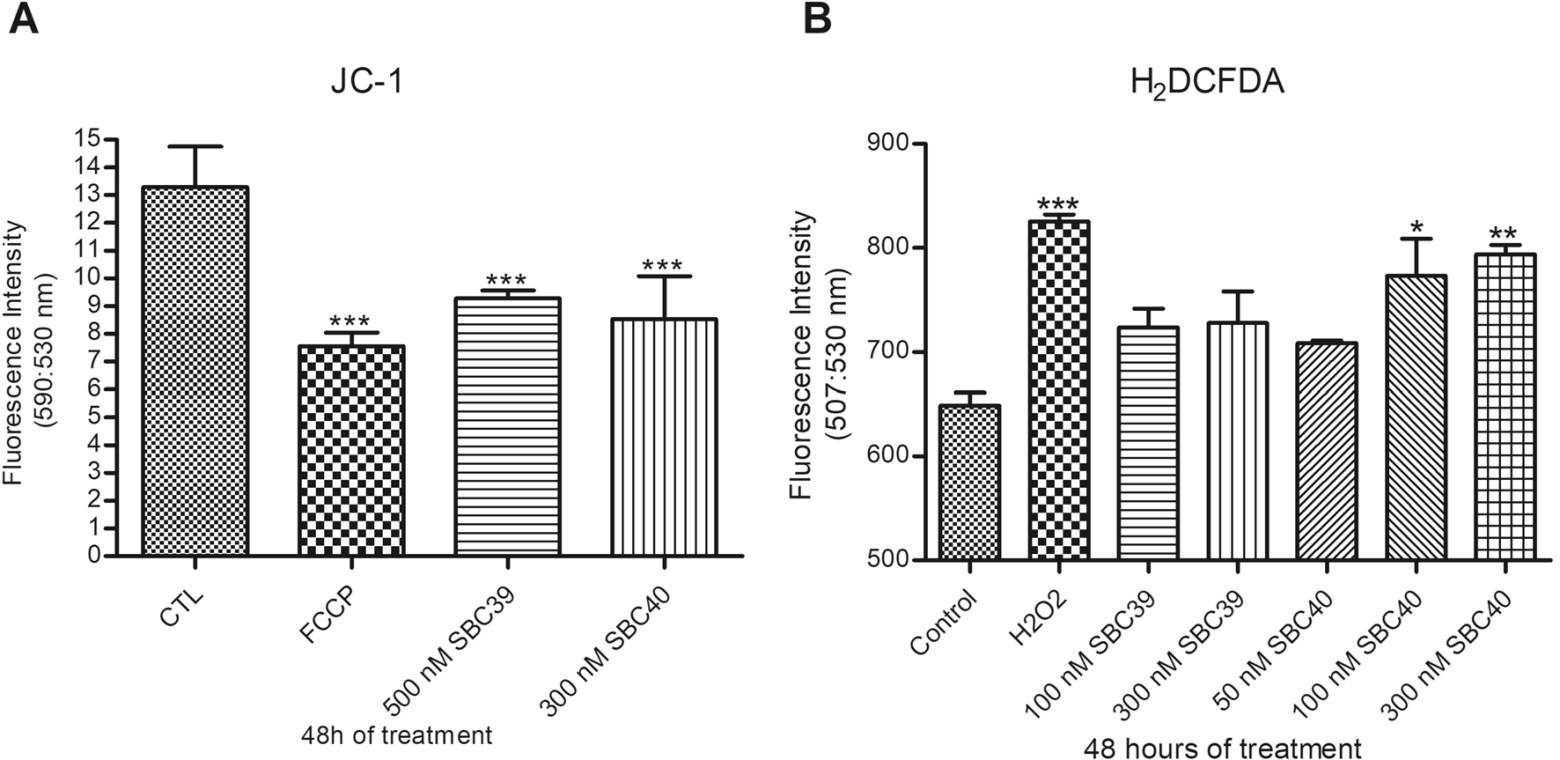
Evaluation of the mitochondrial physiology and function of *L. amazonensis* promastigotes, control and treated with SBC 39 and SBC 40 for 48 h. (**A**) Measurement of Δψ_m_ using JC-1 marker. (**B**) Determination of intracellular ROS by incubating the cells with H_2_DCFDA. The decrease in the Δψ_m_ value indicates a collapse in the mitochondrial transmembrane potential when parasites were treated with SBC 39 and SBC 40. Treatment with 100 nM and 300 nM SBC 40 induced a significant increase in ROS production. FCCP and H_2_O_2_ were used as positive control. The experiments were performed three times in triplicate, and the figures shown represent these three experiments. Bars represent standard deviation. *p < 0.05, **p < 0.01, and ***p < 0.001.

Nanomolar concentrations of SBC 39 and SBC 40 significantly reduced the mitochondrial membrane potential (ΔΨ_*m*_) after 48 h of treatment (Fig. 7A). This effect was similar to those observed in the positive control group treated with FCCP, a mitochondrial protonophore. The analyses were done 25 min after adding JC-1 in all groups when both forms of the fluorochrome (J-monomer and J-aggregate) were stabilized in the inner portion of the mitochondrion. Although both inhibitors reduced the mitochondrial membrane potential, only SBC 40 increased the ROS production significantly at low concentrations (Fig. 7B).

**Figure 8:**
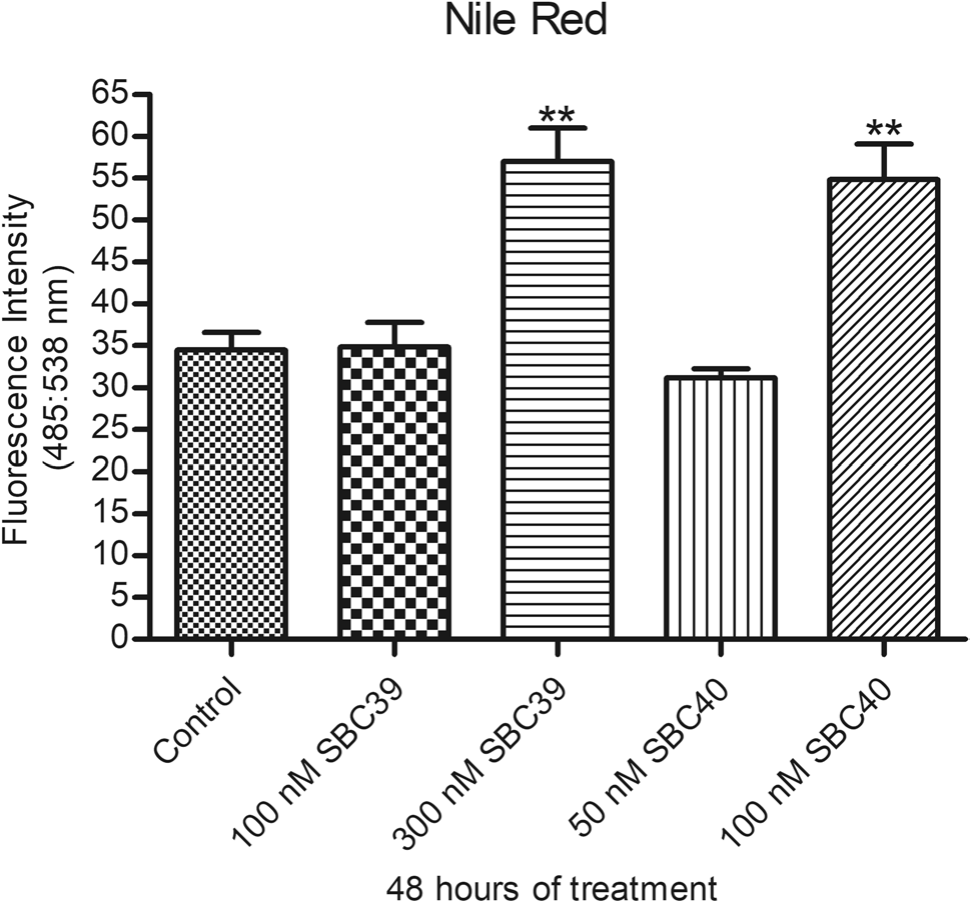
Analysis of Nile Red accumulation. Fluorometric analyses indicate a significant increase in Nile Red accumulation after treatment with 300 nM SBC 39 and 100 nM SBC40. Bars represent standard deviation. *p < 0.05, **p < 0.01, and ***p < 0.001.

Furthermore, both inhibitors could induce lipid bodies accumulation after 48 h of treatment (Fig. 8). However, for SBC 39, the concentration to increase the accumulation of lipid bodies was 3-times higher than those for SBC 40.

### SBC 39 and SBC 40 alter the ultrastructure of promastigotes

**Figure 9:**
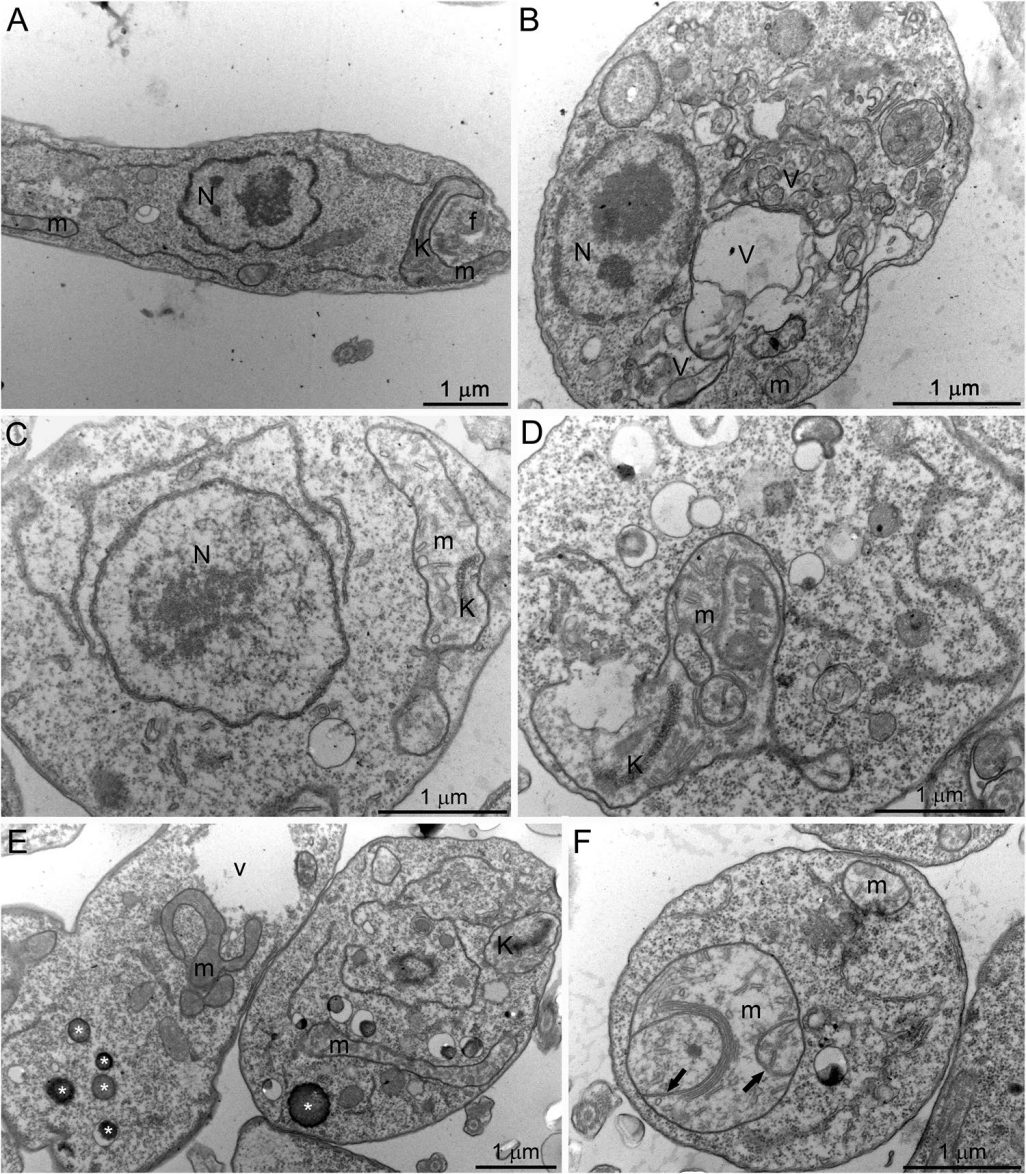
Ultrathin sections of *Leishmania amazonensis* promastigotes, control (**A**) and treated with 500 nM (**B**,**C**) or 1 µM SBC 39 (**D**–**F**) for 48 h. Several alterations were observed, such as: loss of the matrix content and vesiculation of the inner mitochondrial membrane (**C**,**D**,**F**); the presence of several giant vacuoles similar to autophagosomes engulfing portions of the cytoplasm (**B**,**E**); the presence of lipid bodies (**E**); and disorganization of the kinetoplast structure (**C**–**E**). *N* nucleus, *m* mitochondrion, *f* flagellum, *k* kinetoplast, *asterisks* lipid bodies, *v* autophagosome-like vacuole.

**Figure 10:**
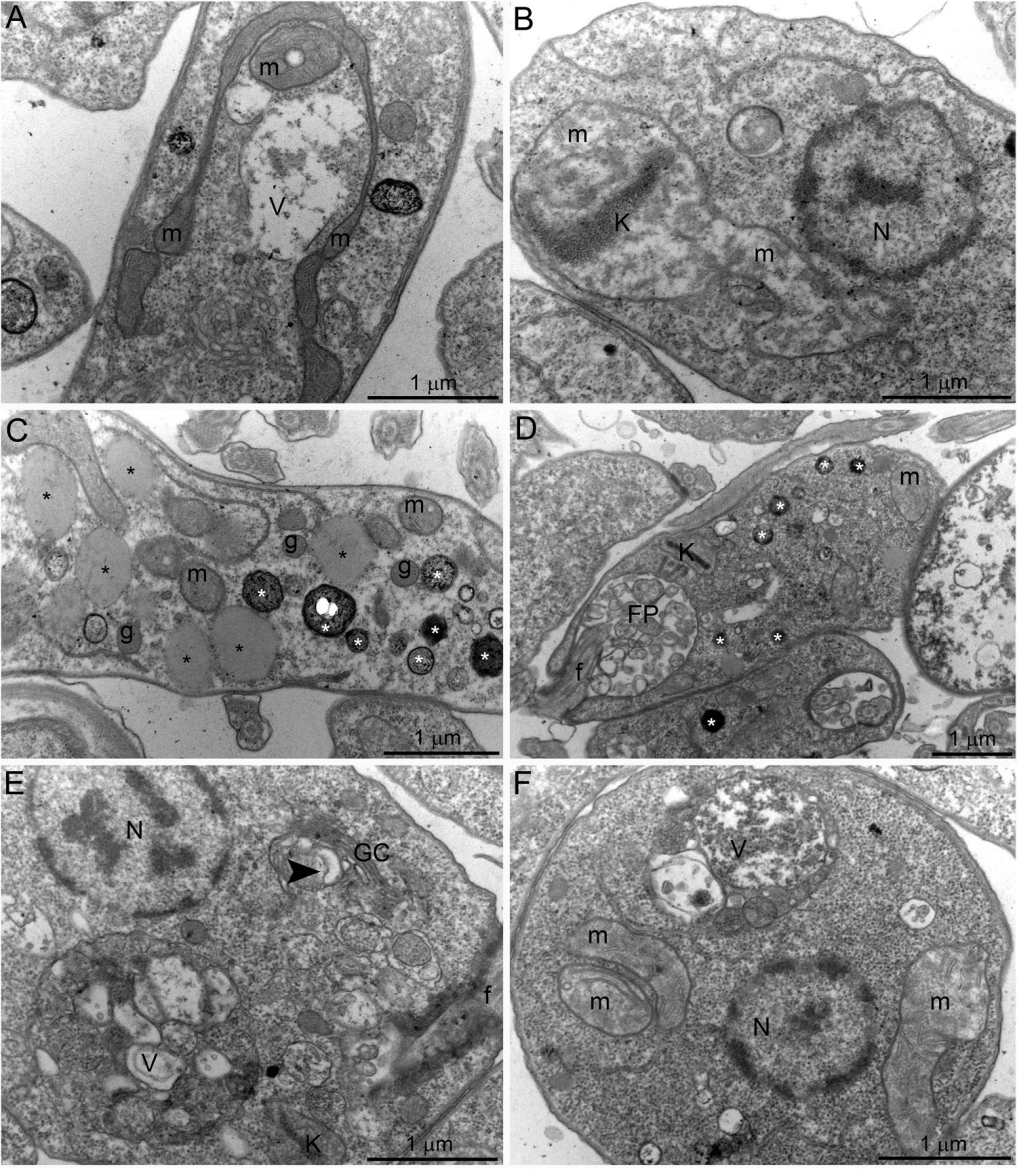
Ultrathin sections of *Leishmania amazonensis* promastigotes treated with 100 nM SBC 40 (**A**–**C**) or 300 nM SBC 40 (**D**–**F**) for 48 h. SBC40 also induced alterations in the parasite ultrastructure such as: mitochondrial swelling and structural disorganization (**A**,**B**,**F**); the presence of many lipid bodies electrondense and eletronlucent (stars, **C**,**D**); a high number of extracellular vesicles inside flagellar pocket (**D**); the presence of several vacuoles similar to autophagosomes containing portions of the cytoplasm and membranes (**A**,**E**,**F**). Panel (**E**) shows a dilation at the trans-Golgi network. *N* nucleus, *m* mitochondrion, *f* flagellum, *GC* Golgi complex, *k* kinetoplast, *asterisks* lipid bodies, *v* autophagosome-like vacuole, *g* glycosome.

Transmission electron microscopy analyzed the ultrastructural alterations induced by SBC 39 and SBC 40. Figure 9A shows a control promastigote presenting a structural organization without any alteration for the nucleus (N), mitochondrion (M), kinetoplast (k), flagellum (f), and cell surface. After 48 h of treatment with 500 nM or 1 µM SBC 39, several alterations were observed, such as: (1) loss of the mitochondrial matrix content and vesiculation of its inner membrane (Fig. 9C,D,F); (2) presence of several large vacuoles similar to autophagosomes engulfing parts of the cytosol (Fig. 9B,E); (3), increased number of lipid bodies (Fig. 9E); and 4) disorganization of the kinetoplast (Fig. 9C–E). For the treatment with SBC 40, in lower concentrations, the ultrastructural alterations were similar to those obtained with SBC 39. In addition, a significant accumulation of lipid bodies randomly distributed throughout the cytosol was observed (Fig. 10C,D). The presence of glycosomes was easily observed in ultrathin sections of treated promastigotes, probably indicating an increasing number of them since they are difficult to observe in control parasites (Fig. 10C). Furthermore, several extracellular vesicles inside the flagellar pocket (Fig. 10D) and the presence of autophagosome-like vacuoles in close association with the nucleus and mitochondrion (Fig. 10A,E,F) were observed. In addition, alterations in the *trans*-Golgi network (Fig. 10E), disorganization of the kinetoplast (Fig. 10B), and intense mitochondrial swelling (Fig. 10B,F) were also induced by the treatments.

**Figure 11:**
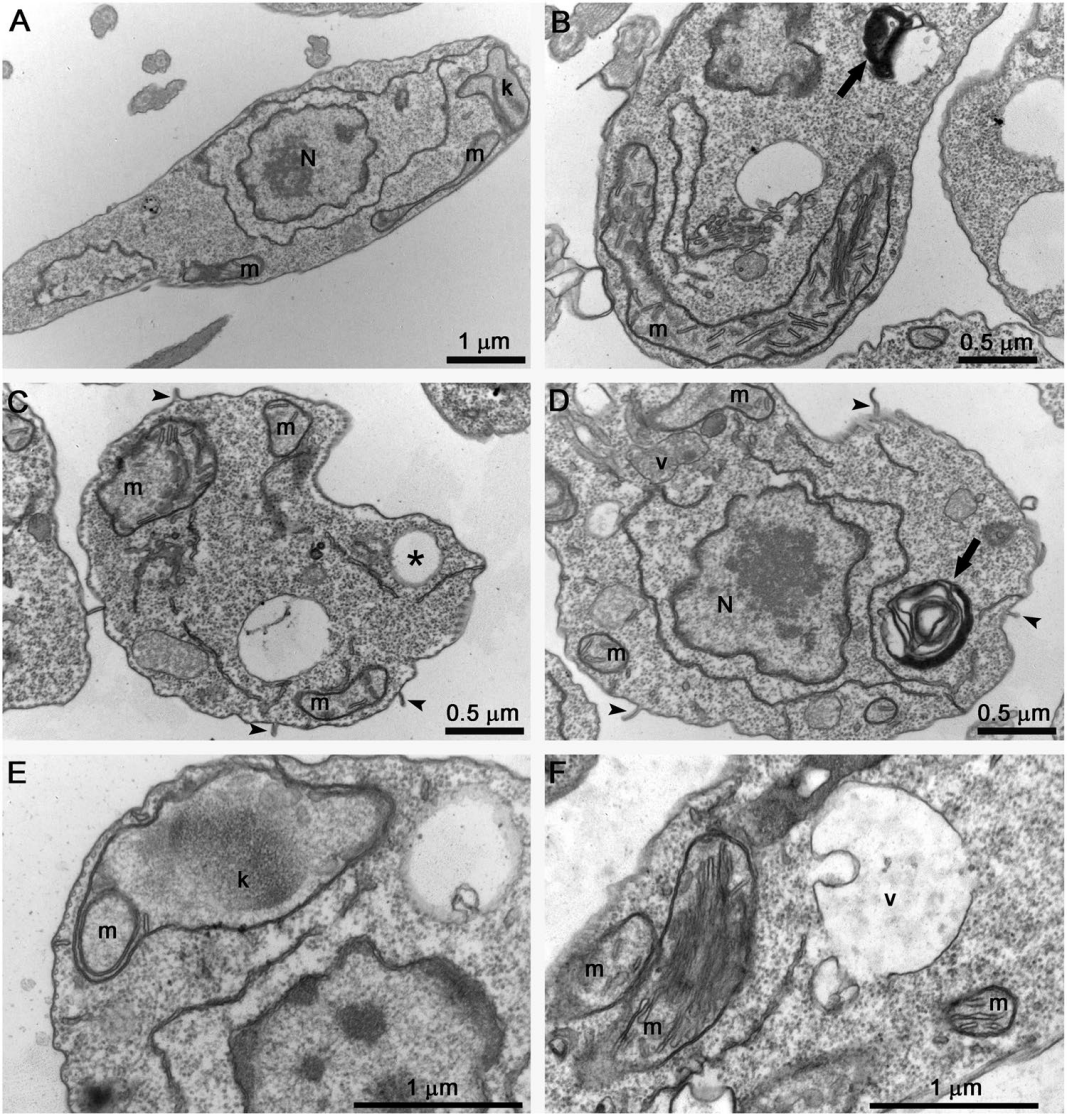
Ultrathin sections of *Leishmania amazonensis* promastigotes, control (**A**) and treated with 5 µM SBC 39 (**B**–**D**) or SBC 40 (**E**,**F**) for 6 h. After a short time of incubation with the compounds, promastigotes also presented several alterations such as: mitochondrion swelling with a significant increased number of cristae (**B**,**C**,**E**,**F**); plasma membrane projections (**B**–**D**, arrowheads); disorganization of the kinetoplast (**E**); and presence of large vacuoles and a myelin like-figure in close association with the endoplasmic reticulum (**B**,**C**,**E**,**F**, arrows). *N* nucleus, *m* mitochondrion, *f* flagellum, *k* kinetoplast, *asterisk* lipid body, *v* vacuole.

**Figure 12:**
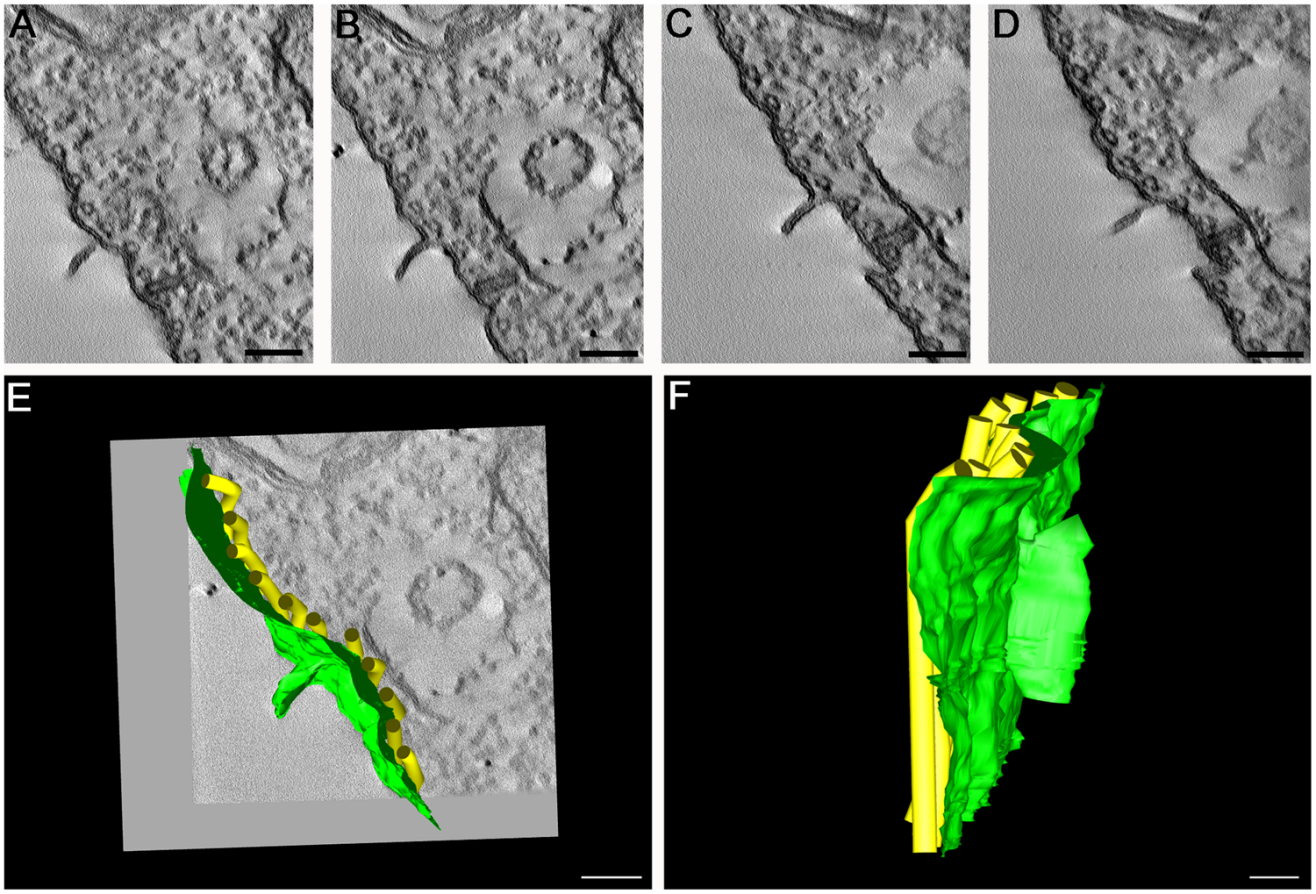
Electron microscopy tomography and 3D reconstruction showing a projection of the plasma membrane of *L. amazonensis* promastigotes treated with 5 µM SBC 39 for 6 h. The images confirmed the presence of the endoplasmic reticulum profile near the projection and the absence of microtubules associated with it.

To better understand the ultrastructural effects induced by SBs, we decided to carry out treatments with high concentrations and a short incubation time. For this, we used a concentration of 5 µM for just 6 h. The results obtained indicated significant alterations showing that these new compounds presented a potent activity against *Leishmania amazonensis.* After treatment, plasma membrane projections were observed with 5 µM SBC 39 (Fig. 11B–D, arrowhead). Interestingly, these projections appeared frequently in regions of the membrane close to the endoplasmic reticulum (Fig. 12D, arrowhead). Therefore, we decided to observe these projections by electron tomography. Figure 12A–D shows a serial section tomography of *L. amazonensis* promastigotes treated with 5 µM SBC 39 for 6 h. From these sections, a specific area of the parasite surface was reconstructed (Fig. 12E,F), revealing the ultrastructure of this plasma membrane projection. Images confirmed the presence of the endoplasmic reticulum profile near the projection and the absence of microtubules associated with it. The alignment of the electron tomograms allows us to observe the relationship between the projection and the endoplasmic reticulum (Suppl. Figures 1 and 2—movies).

Furthermore, the treatment with SBC 39 and SBC 40 for a short time caused several other alterations in the ultrastructure of the promastigotes, such as: mitochondrial swelling with an increased number of mitochondrial cristae (Fig. 11B,C,F); the presence of large vacuoles and myelin-like figures (Fig. 11B–F); and disorganization of the kinetoplast (Fig. 12E). After the alignment of several electron tomograms, it is possible to observe some of these changes in a movie containing a large volume of one promastigote treated with 5 µM SBC 39 (Suppl. Figure 3—movie).

### Effects of SBC 40 on the fine structure of intracellular amastigotes

**Figure 13:**
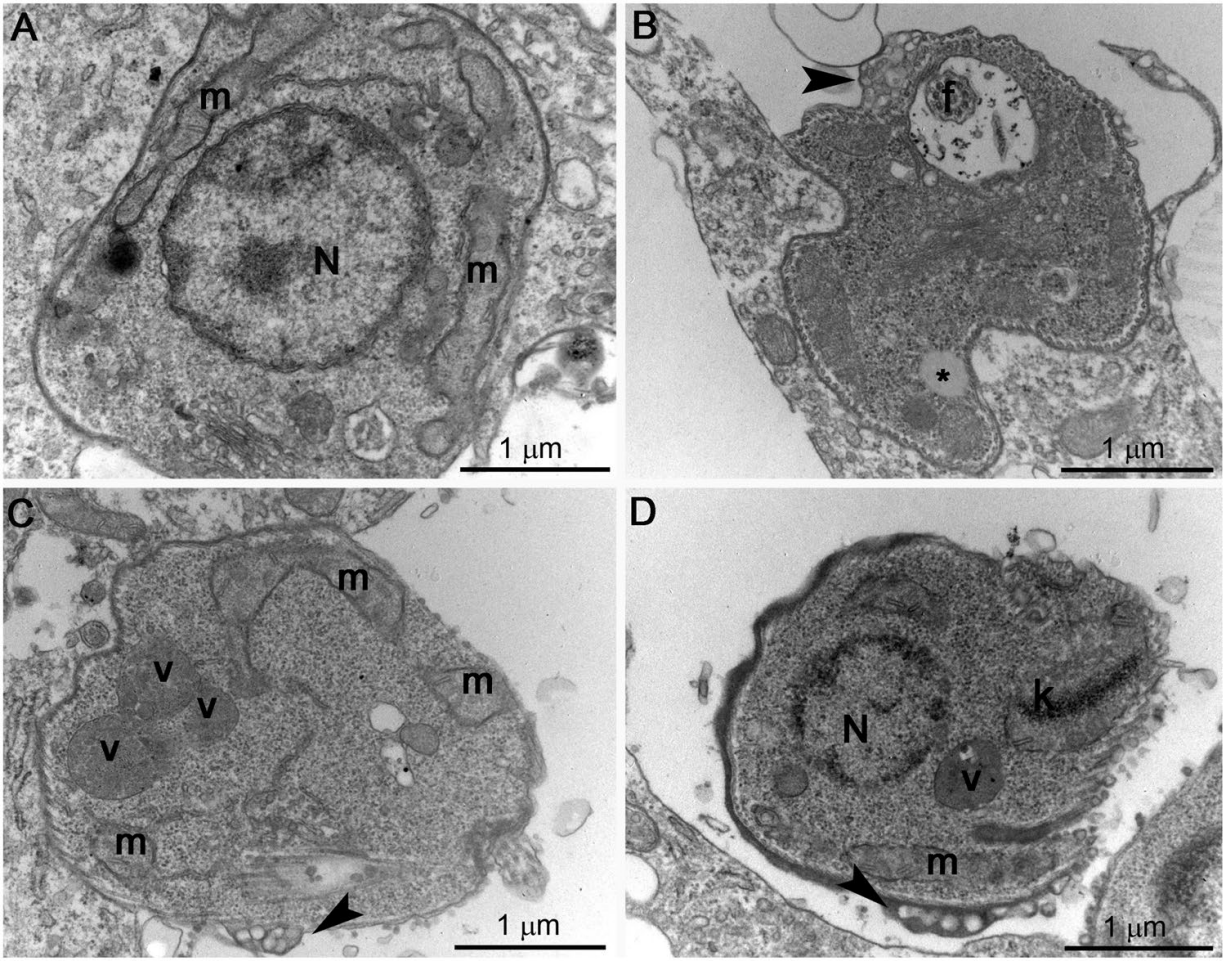
Ultrathin section of *L. amazonensis* intracellular amastigotes treated with 1 µM of SBC 40 for 48 h. Parasites treated with SBC40 appeared with drastic alterations in the plasma membrane (**B**–**D**, arrowhead), mitochondrial swelling (**C**), presence of autophagosome-like vacuoles (**C**,**D**), and disorganization of the kinetoplast (**D**). *N* nucleus, *m* mitochondrion, *f* flagellum, *k* kinetoplast, *v* autophagosome-like vacuole.

Transmission electron microscopy was also used to study the fine structure of intracellular amastigotes and the effects induced by the treatments. Figure 13 shows the ultrathin sections of *L. amazonensis* intracellular amastigotes treated with 1 µM SBC 40 for 48 h. Several alterations in the ultrastructure were observed, which are absent in the control parasites (Fig. 13A), such as: mitochondrial swelling (Fig. 13C); the presence of autophagosome-like vacuoles containing membrane profiles (Fig. 13C,D); alterations in the plasma membrane ultrastructure (Fig. 13B–D, arrowhead); and disorganization of the kinetoplast (Fig. 13D). Together, these images suggest the potent effect of benzyl farnesyl amine mimetics in *Leishmania amazonensis* intracellular amastigotes.

### Identification and quantification of the free sterol

**Figure 14:**
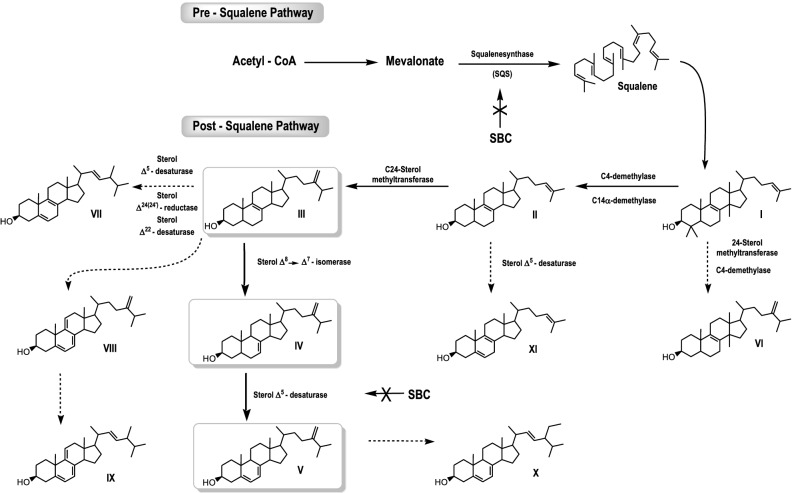
Sterol biosynthesis pathway in *L. amazonensis* from the sterols identified in the current study. (I) 4,4′,14-trimethyl-cholesta-8,24-dien-3β-ol (lanosterol); (II) cholesta-8,24-dien-3β-ol (zymosterol); (III) ergosta-8,24(24′)-dien-3β-ol (fecosterol); (IV) ergosta-7,24(24′)-dien-3β-ol (episterol); (V) ergosta-5,7,24(24′)-dien-3β-ol (5-dehydroepisterol); (VI) 14-methyl-ergosta-8,24(24′)-dien-3β-ol; (VII) ergosta-5,8,22-trien-3β-ol; (VIII) ergosta-5,7,9(10)-24(24′)-tetraen-3β-ol; (IX) ergosta-5,7,9(10)-22-tetraen-3β-ol; (X) stigmasta-5,7,22-trien-3β-ol; (XI) cholesta-5,8,24-trien-3β-ol. Thick lines indicate main reaction pathways. Dashed arrows show intermediate sterols of lower quantity. Arrows marked with an ‘X’ indicate inhibitory interactions of the SBC analogs.

Analyses of the free sterol composition of control and treated *Leishmania amazonensis* promastigotes (Table 1) (Fig. 14) revealed that the major sterols of control (untreated) promastigotes were ergosta-5,7,24(24′)-trien-3β-ol (5-dehydroepisterol) and ergosta-7,24(24′)-dien-3β-ol (episterol), both synthesized de novo, which accounted for 68% and 12%, respectively, of the total sterols. While other minority sterols such as zymosterol, cholesta-5,8,24-trien-3β-ol, ergosta-5,7,9(11)-22-tetraen-3β-ol, ergosta-5,8,22-trien-3β-ol, 14α-methyl-ergosta-8,24(24′)-dien-3β-ol, ergosta-8,24(24′)-trien-3β-ol, ergosta-5,7,9(10)-24(24′)-tetraen-3β-ol, lanosterol, stigmasta-5,7,22-trien-3β-ol, stigmasta-7,22-trien-3β-ol were detected, and the sum of them represented 14% (Table 1). Additionally, cholesterol, taken by endocytosis from the growth medium, accounted for 6%.

**Table 1:** Composition of free sterols from SBC analogs-treated and non-treated *L. amazonensis* promastigotes.

Sterol detected*	RT (min)	Control	SBC39 300 nM	SBC39 500 nM	SBC39 1.0 µM	SBC40 30 nM	SBC40 100 nM	SBC40 300 nM
Cholesterol	22.40	6.0	8.2	11.3	19.1	7.0	17.2	25.9
Zymosterol (II)	23.20	0.8	nd	nd	nd	nd	nd	nd
Cholesta-5,8,24-trien-3β-ol (XI)	24.07	1.3	nd	nd	nd	nd	nd	nd
Ergosta-5,7,9(11)-22-Tetraen-3β-ol (IX)	24.61	1.6	nd	nd	nd	1.9	3.0	4.0
Ergosta-5,8,22-trien-3β-ol (VII)	24.88	nd	2.7	3.1	nd	nd	1.7	nd
14α-Methyl-ergosta-8,24(24′)-dien-3ß-ol (VI)	24.90	2.5	nd	nd	nd	1.7	nd	nd
Ergosta-8,24(24′)-dien-3β-ol (III)	25.05	1.2	41.7	42.9	39.5	nd	11.5	16.0
5-Dehydroepisterol (V)	25.9	67.9	23.2	24.1	30.2	65.9	35.8	36.7
Episterol (IV)	26.19	12.2	21.7	15.1	7.9	16.7	25.3	15.5
Ergosta-5,7,9(10)-24(24′)-tetraen-3β-ol (VIII)	26.36	3.0	nd	nd	nd	2.1	1.3	nd
Lanosterol (I)	27.43	2.1	2.7	3.4	3.3	2.9	2.5	2.0
Stigmasta-5,7,22-trien-3β-ol (X)	28.37	1.2	nd	nd	nd	1.8	1.7	nd

The numbers found in the columns for control and treated parasites represent the percentage of total sterols.

*RT* retention time, *nd* no detected; Sterols structure: see Fig. 13.

Incubation of *L. amazonensis* promastigotes with increasing concentrations of SBC 39 (300 nM–1 µM) or SBC 40 (30 nM–300 nM) for 48 h induced reduction of the relative level of the main endogenous sterol 5-dehydroepisterol in about 50%. However, promastigotes in the presence of SBC 39 displayed a significant accumulation of ergosta-8, 24(24′)-dien-3β-ol (fecosterol) to 40%, while in the presence of the highest concentration of SBC 40, fecosterol reached only 16% (Table 1) (Fig. 14). Episterol is not significantly affected. These changes in the relative percentages of endogenous sterols are compensated with an increase in the percentage of cholesterol, which accounts for ca. 19% and 26% for SBC 39 and SBC 40, respectively.

When performing the quantitative analysis of cholesterol and endogenous sterols (Table 2), both drugs (SBC 39 and SBC 40) induce a concomitant reduction in the content of endogenous sterols compared to untreated cells. For instance, SBC 40 at concentrations of 30 nM and 300 nM reduced endogenous sterols to 464.5 and 68.1 ng/108 cells, reducing approximately 40.6% and 91.3%, respectively, compared to 781 ng/10^8^ contained in control cells. Likewise, the cholesterol/endogenous sterol ratio of the control cells (1:43) increased (1/33; 1/12; 1/7) as the concentration of SBC 40 increased, clearly showing the effect on the synthesis of sterols (Table 2).

**Table 2:** Cholesterol and endogenous sterols^a^ contents in control and SBC-treated *L. amazonensis* promastigotes.

Sterol measured	Control	SBC39 300 nM	SBC39 500 nM	SBC39 1000 nM	SBC40 30 nM	SBC40 100 nM	SBC40 300 nM
		Mean (ng/1 × 10^8^ cell) (SD)					
Cholesterol	18.0 (0.3)	13.7 (0.4)	11.4 (0.4)	9.3 (0.4)	13.9 (0.3)	10.9 (0.3)	9.7 (0.4)
Endogenous sterols	781.0 (61.2)	369.0 (70.3)	220.6 (91.9)	96.8 (89.6)	464.5 (51.6)	126.8 (70.4)	68.1 (71.7)
Ratio^b^	1:43	1:27	1:19	1:10	1:33	1:12	1:7

Mean and standard deviations (SD) were calculated from three parallel measurements (*n* = 3).

^a^Endogenous sterols = Ergosta-8,24(24′)-trien-3β-ol, 5-dehydroepisterol and episterol.

^b^The cholesterol:endogenous sterols ratio.

## Discussion

Although leishmaniasis has several treatment options, its therapy has a lot of problems such as extensive toxicity, lack of efficacy, parenteral route of administration affecting compliance, high costs, and emerging drug resistance^29^. Moreover, visceral, cutaneous, and mucocutaneous leishmaniasis remains some of the most devastating neglected tropical diseases. Thus, there is an urgent need to develop new anti-leishmanial compounds with more efficacy, low toxicity, and cost, which are preferentially administrated by oral or topic routes.

Sterol biosynthesis (SB) is an essential metabolic pathway in *Leishmania* sp. For many years, several SB inhibitors have been studied against both developmental stages of the parasite in vitro^15–17,23,28,30–32^. Some of them have also been tested in vivo against *Trypanosoma cruzi*, inducing a potent suppressive effect in acute Chagas’ disease^30,33–35^. An important step of the SB is catalyzed by the enzyme squalene synthase (SQS). SQS is responsible for the reaction that catalyzes the first committed step in the SB pathway, thus, not interfering with isoprenoid production and its metabolites^11,36^. Several squalene synthase (SQS) inhibitors, such as quinuclidine derivatives, have been studied as potent SB inhibitors. The first quinuclidine derivatives tested against *Leishmania* sp. were BPQ-OH, ER-119884, and E5700, inducing cell death associated with the depletion of the parasite’s endogenous sterols^23,31^. Furthermore, another quinuclidine derivative, WSP 1267, showed a potent effect against *Candida albicans*, *C. parapsilosis*, and *C. tropicalis*, with a MIC_50_ of 2 µg/ml^37^. Some quinuclidine derivatives were also able to inhibit the recombinant *L. major* SQS at submicromolar concentrations, exhibiting selectivity action for the parasite enzyme^20,21^. Benzyl farnesyl amine mimics were also reported to be selective inhibitors of human SQS. They have been explored for their potential use in developing cholesterol-lowering treatment options for hypercholesterolemia in men^38^.

In this study, we report the activity of several benzyl farnesyl amine mimetics against *Leishmania amazonensis*. The compounds SBC 39 and SBC 40 showed the most pronounced effects on the growth of *L. amazonensis* intracellular amastigotes, associated with low cytotoxicity in mammal cells and a higher selectivity index of 117.7 and 180, respectively. These selectivity indexes are higher than those found after treatment with posaconazole and itraconazole, two potent inhibitors of the growth of *L. amazonensis*^16^. Herein, the antiproliferative activities of SBC 39 and SBC 40 were in the nanomolar range against both extracellular promastigotes and intracellular amastigotes. Furthermore, the biological activity against *Leishmania amazonensis* was lower than those found for E5700 and ER-119884, two SQS inhibitors from Eisai Pharmaceutical Company, previously studied against *Leishmania*^17,26^. Nevertheless, it is noteworthy to mention that further development of E5700 and ER 119884 has been stopped because it caused testicle atrophy in a small animal experiment (personal communication). Beyond this, quinuclidine derivatives often bear a certain risk of neurological side effects (neurotoxicity of antimalarial drug quinine and other quinuclidine-containing drugs). Therefore, medicinal chemistry is increasingly interested in avoiding the quinuclidine moiety in early phase drug development. Finally, benzyl farnesyl amine mimetics have a much lower production cost, which is especially highly attractive for anti-infectious drug design in tropical emerging countries, where cost restrictions sometimes limit pharmaceutical development. Thus, these results should be regarded as a precious contribution to SB inhibitors research in tropical parasites with a high potential for further drug development.

We used scanning and transmission electron microscopy to analyze morphological and ultrastructural alterations. Some morphological alterations such as the swelling of the cell body and the appearance of cells with several flagella were observed. Although the number of flagella in treated promastigotes was altered, arrest of the cell cycle was not observed, which is also found for other SB inhibitors^16,17,28^. Transmission electron microscopy images indicated that mitochondrion ultrastructure was dramatically altered after treatment with SBC 39 and SBC 40 (Figs. 9, 10). Fluorescence markers for mitochondrial membrane potential and ROS production confirmed the mitochondrial damage provoked by the treatments (Fig. 7). Alterations in the mitochondrion structure and function could be related to significant changes in the lipidic composition of the mitochondrial membranes since previous studies showed that the unique and ramified mitochondrion of the trypanosomatid has a particular composition of 24-methyl sterols^14^. Interestingly, in some images, several glycosomes were observed after treatment with SBC 40 (Fig. 10C), which could indicate an effort of the treated parasite to supply the mitochondrial damages and a possible decrease in the oxidative phosphorylation. An increasing number of glycosomes could help the parasite compensate for ATP production using the glycolytic pathway.

Another important alteration was observed in the plasma membrane (Fig. 6), which could result from 24-methyl sterols depletion replaced by toxic intermediates of the sterol biosynthesis. This phenotype was also observed after treatment with several other SB inhibitors in *Leishmania*^15–17,26,27,38^. Moreover, several projections on the plasma membrane were observed after only 6 h of treatment, which was better observed by electron tomography (Figs. 11, 12). Projections could also be related to the secretory pathway since in some images, as shown in Fig. 10D, we observed several extracellular vesicles secreted by treated promastigotes. In addition, the presence of several giant vacuoles containing portions of the cytosol, damaged organelles, and membranes that could be related to an intense autophagic process, also indicating an increase in the secretory activity by the treated promastigotes.

Using GC–MS, we documented the relative composition (%) of eleven sterols in the membranes of *Leishmania amazonensis* promastigotes (Table 1). In our study, the major sterol detected were episterol (IV) and 5-dehydroepisterol (V). Nevertheless, less usual sterols were also observed to a lesser degree, including cholesta-5,8,24-trien-3β-ol (XI), ergosta-5,7,9(10)-24(24′)-tetraen-3β-ol (VIII), ergosta-5,7,9(11)-22-tetraen-3β-ol, and (IX) ergosta-5,8,22-trien-3β-ol (VII). In fungi, few reports exist describing the detection of this kind of sterols, for which no role has been assigned yet^39,40^. Moreover, the accumulation of ergosta-8,24(24′)-dien-3β-ol (III) (fecosterol) in cells treated with both SBC 39 and SBC 40 indicated that these inhibitors not only impair the activity of the ∆^8^ → ∆^7^ double-bond isomerization (C-8 sterol isomerase/ERG2), but also the formation of a double bond between C-5 and C-6 in the B ring of sterols (C-5 sterol desaturase/ERG3).

Furthermore, the results (Tables 1, 2) indicated that both compounds (SBC 39 and 40) caused a concentration-dependent reduction in the ratio of endogenous 24-alkyl sterols (fecosterol, episterol, and 5-dehydroepisterol) to cholesterol (exogenous) below a critical level as in previous studies involving other SQS inhibitors^17,23^. For instance, the ratio of cholesterol content to endogenous sterols in promastigotes control was 1:43. In contrast, in promastigotes treated with SBC 40 (at 300 nM), the cholesterol to endogenous sterols ratio changed dramatically to 1:7. The result is consistent with a blockade of de novo sterol synthesis at the level of SQS.

Thus, the results suggest that both SBC 39 and SBC 40 have potent antiproliferative and selective growth inhibition effects on *Leishmania amazonensis,* and remarkably, they both have a dual mode of action (Fig. 14). The first is its interference in de novo sterol biosynthesis at the SQS level (Pre-Squalene Pathway); the second is the inhibition of downstream sterol biosynthesis, specifically in the isomerization of the ∆8 → ∆7 ***double bond and in the formation of the double bond between C-5 and C-6 of the ring B of the sterol (Post- Squalene Pathway).

In conclusion, our results support the notion that SBC 39 and SBC 40 are promising new chemotherapeutic agents against *Leishmania* sp. since they presented a very high specificity for the parasite. Furthermore, our findings justify future studies to better understand the mode of action and use in combination therapy with other SB inhibitors as a new therapeutic strategy that could reduce toxicity but increase the efficacy of treatment.

## Supplementary Information

Supplementary Information 1.

Supplementary Video 1.

Supplementary Video 2.

Supplementary Video 3.

## References

[1] Alvar J (2012). Leishmaniasis worldwide and global estimates of its incidence. PLoS One.

[2] Oryan A, Akbari M (2016). Worldwide risk factors in leishmaniasis. Asian Pac. J. Trop. Med..

[3] Silveira FT, Lainson R, de Castro Gomes CM, Laurenti MD, Corbett CE (2009). Immunopathogenic competences of *Leishmania* (V.) *braziliensis* and L. (L.) amazonensis in American cutaneous leishmaniasis. Parasite Immunol..

[4] Silveira FT, Lainson R, Corbett CE (2005). Further observations on clinical, histopathological, and immunological features of borderline disseminated cutaneous leishmaniasis caused by *Leishmania (Leishmania) amazonensis*. Mem. Inst. Oswaldo Cruz.

[5] Davidson RN (1998). Practical guide for the treatment of leishmaniasis. Drugs.

[6] Uliana SR, Trinconi CT, Coelho AC (2017). Chemotherapy of leishmaniasis: Present challenges. Parasitol.

[7] Croft SL, Neal RA, Pendergast W, Chan JH (1987). The activity of alkyl phosphorylcholines and related derivatives against *Leishmania donovani*. Biochem. Pharmacol..

[8] Sundar S (2002). Oral miltefosine for Indian visceral leishmaniasis. N. Eng. J. Med..

[9] Croft SL, Sundar S, Fairlamb AH (2006). Drug resistance in leishmaniasis. Clin. Microbiol. Rev..

[10] van Griensven J (2010). Combination therapy for visceral leishmaniasis. Lancet Infect. Dis..

[11] de Macedo-Silva ST, de Souza W, Rodrigues JCF (2015). Sterol biosynthesis pathway as an alternative for the antiprotozoan parasite chemotherapy. Curr. Med. Chem..

[12] Shang N (2014). Squalene synthase as a target for Chagas disease therapeutics. PLoS Pathog..

[13] Hargrove TY (2013). Complexes of *Trypanosoma cruzi* sterol 14α-demethylase (CYP51) with two pyridine-based drug candidates for Chagas disease: Structural basis for pathogen selectivity. J. Biol. Chem..

[14] Rodrigues JC, de Souza W (2008). Ultrastructural alterations in organelles of parasitic protozoa induced by different classes of metabolic inhibitors. Curr. Pharm. Des..

[15] de Macedo-Silva ST, de Oliveira Silva TL, Urbina JA, de Souza W, Rodrigues JCF (2011). Antiproliferative, ultrastructural, and physiological effects of amiodarone on promastigote and amastigote forms of *Leishmania amazonensis*. Mol. Biol. Int..

[16] de Macedo-Silva ST, Urbina JA, de Souza W, Rodrigues JCF (2013). In vitro activity of the antifungal azoles itraconazole and posaconazole against *Leishmania*. PLoS One.

[17] de Macedo-Silva ST, Visbal G, Urbina JA, de Souza W, Rodrigues JCF (2015). Potent in vitro antiproliferative synergism of combinations of ergosterol biosynthesis inhibitors against *Leishmania amazonensis*. Antimicrob. Agents Chemoth..

[18] Abe I, Tomesch JC, Wattanasin S, Prestwich GD (1994). Inhibitors of squalene biosynthesis and metabolism. Nat. Prod. Rep..

[19] Kourounakis AP, Katselou MG, Matralis AN, Ladopoulou EM, Bavavea E (2011). Squalene synthase inhibitors: An update on the search for new antihyperlipidemic and antiatherosclerotic agents. Curr. Med. Chem.

[20] Cammerer SB (2007). Quinuclidine derivatives as potential antiparasitics. Antimicrob. Agents Chemoth..

[21] Orenes Lorente S (2005). Biphenylquinuclidines as inhibitors of squalene synthase and growth of parasitic protozoa. Bioorg. Med. Chem..

[22] Martins-Duarte ES, Urbina JA, de Souza W, Vommaro RC (2006). Antiproliferative activities of two novel quinuclidine inhibitors against *Toxoplasma gondii* tachyzoites *in vitro*. J. Antimicrob. Chemoth..

[23] Rodrigues JCF (2008). In vitro activities of ER-119884 and E5700, two potent squalene synthase inhibitors, against *Leishmania amazonensis*: Antiproliferative, biochemical, and ultrastructural effects. Antimicrob. Agents Chemoth..

[24] Warren LG (1960). Metabolism of Schizotrypanum cruzi Chagas. I. Effect of culture age and substrate concentration on respiratory rate. J. Parasitol..

[25] Souza GF, Cämmerer SB, Franco CH, Moraes CB, Freitas-Junior LH (2016). *N-*[4-[Benzyloxy]benzyl]-benzenemethaneamines with high biological activity against intracellular *Trypanosoma cruzi* and *Leishmania infantum* amastigotes. Int. J. Chem. Pharm. Sci..

[26] Godinho JL, Georgikopoulou K, Calogeropoulou T, de Souza W, Rodrigues JCF (2013). A novel alkyl phosphocholine-dinitroaniline hybrid molecule exhibits biological activity in vitro against *Leishmania amazonensis*. Exp. Parasitol..

[27] Kremer JR, Mastronarde DN, Mclntosh JR (1996). Computer visualization of three-dimensional image data using IMOD. J. Struct. Biol..

[28] de Macedo Silva ST (2018). In vitro antileishmanial activity of ravuconazole, a triazole antifungal drug, as a potential treatment for leishmaniasis. J. Antimicrob. Chemother..

[29] Zulfiqar B, Shelper TB, Avery VM (2017). Leishmaniasis drug discovery: Recent progress and challenges in assay development. Drug Discov. Today.

[30] Urbina JA, Concepcion JL, Rangel S, Visbal G, Lira R (2002). Squalene synthase as a chemotherapeutic target in a *Trypanosoma cruzi* and *Leishmania*. Mol. Biochem. Parasitol..

[31] Rodrigues JCF, Urbina JA, de Souza W (2005). Antiproliferative and ultrastructural effects of BPQ-OH, a specific inhibitor of squalene synthase, on *Leishmania amazonensis*. Exp. Parasitol..

[32] Rodrigues JCF, Attias M, Rodriguez C, Urbina JA, de Souza W (2002). Ultrastructural and biochemical alterations induced by 22,26-azasterol, a Δ24(25)-sterol methyltransferase inhibitor, on promastigote and amastigote forms of *Leishmania amazonensis*. Antimicrob. Agents Chemother..

[33] Diniz LF, Caldas IS, Guedes PM, Crepalde G, de Lana M, Carneiro CM, Talvani A, Urbina JA, Bahia MT (2010). Effects of ravuconazole treatment on parasite load and immune response in dogs experimentally infected with *Trypanosoma cruzi*. Antimicrob. Agents Chemother..

[34] Urbina JA, Payares G, Contreras LM, Liendo A, Sanoja C, Molina J, Piras M, Piras R, Perez N, Wincker P, Loebenberg D (1998). Antiproliferative effects and mechanism of action of SCH 56592 against *Trypanosoma (Schizotrypanum) cruzi*: In vitro and in vivo studies. Antimicrob. Agents Chemother..

[35] Papadopoulou MV, Bloomer WD, Rosenzweig HS, O’Shea IP, Wilkinson SR, Kaiser M, Chatelain E, Ioset JR (2015). Discovery of potent nitrotriazole-based antitrypanosomal agents: In vitro and in vivo evaluation. Bioorg. Med. Chem..

[36] Brown GR, Clarke DS, Foubister AJ, Freeman S, Harrison PJ, Johnson MC, Mallion KB, McCormick J, McTaggart F, Reid AC, Smith GJ, Taylor MJ (1996). Synthesis and activity of a novel series of 3-biarylquinuclidine squalene synthase inhibitors. J. Med. Chem..

[37] Ishida K, Rodrigues JCF, Cammerer S, Urbina JA, Gilbert I, de Souza W, Rozental S (2011). Synthetic arylquinuclidine derivatives exhibit antifungal activity against *Candida albicans*, *Candida tropicalis* and *Candida parapsilopsis*. Ann. Clin. Microbiol. Antimicrob..

[38] Brinkmann JA, Damon RE, Fell JB, Perez LB, Scallen TJ, Vedananda TR (1996). Squalene synthase inhibitors: Isosteric replacements of the farnesyl side chain of benzyl farnesyl amine. Bioorg. Med. Chem. Lett..

[39] Ruan B, Lai PS, Yeh CW, Wilson WK, Pang J, Xu R, Matsuda SPT, Schroepfer GJ (2002). Alternative pathways of sterol synthesis in yeast. Use of C27 sterol tracers to study aberrant double-bond migrations and evaluate their relative importance. Steroids.

[40] Alcazar-Fouli L, Mellado E, Garcia-Effron G, Lopez JF, Grimalt JO, Cuenca-Estrella JM, Rodriguez-Tudela JL (2008). Ergosterol biosynthesis pathway in *Aspergillus fumigatus*. Steroids.

