# Supplementary material for: Benzylamines as highly potent inhibitors of the sterol biosynthesis pathway in Leishmania amazonensis leading to oxidative stress and ultrastructural alterations
Source: Sci Rep. 2022 Jul 4;12:11313. doi: 10.1038/s41598-022-15449-3 (PMC9253131; doi:10.1038/s41598-022-15449-3)
Supplement: Supplementary file 1 — Supplementary Information 1. [file 41598_2022_15449_MOESM1_ESM.docx]

**Legend to Supplementary Material.**

**Supp. Fig. 1. Movie 1.** Movie obtained after 3D reconstruction of a promastigote treated with SBC 39 to observe a plasma membrane projection and its relationship with the endoplasmic reticulum.

**Supp. Fig. 2. Movie 2.** Another movie obtained after 3D reconstruction of a promastigote treated with SBC 39 to observe a plasma membrane projection and its relationship with the endoplasmic reticulum. It is possible to observe the absence of subpellicular microtubule associated with the projection.

**Supp. Fig. 3.** Movie 3. 3D reconstruction of a large volume of one promastigote treated with 5 µM SBC 39 showing a large vacuole containing internal membrane and a myelin-like figure. We also observe several mitochondrial cristae.
